# GIMAP7 inhibits epithelial‐mesenchymal transition and glycolysis in lung adenocarcinoma cells via regulating the Smo/AMPK signaling pathway

**DOI:** 10.1111/1759-7714.15150

**Published:** 2023-12-27

**Authors:** Liyuan Cui, Yumei Shen, Shanzhou Duan, Qifeng Ding, Yifei Wang, Wentao Yang, Yongbing Chen

**Affiliations:** ^1^ Department of Thoracic Surgery The Second Affiliated Hospital of Soochow University Suzhou China; ^2^ Operation Room Department The Second Affiliated Hospital of Soochow University Suzhou China

**Keywords:** epithelial‐mesenchymal transition, GIMAP7, glycolysis, lung adenocarcinoma, Smo/AMPK signaling

## Abstract

**Background:**

GTPase immunity‐associated protein 7 (GIMAP7) has been previously recognized as a prognostic marker in pan‐cancer. Our objective was to explore the function of GIMAP7 in the progression of lung adenocarcinoma (LUAD).

**Methods:**

GIMAP7 was overexpressed by transfection with GIMAP7 plasmid, and knocked down using siRNAs. The biological functions of GIMAP7 were examined by employing CCK‐8, EdU, colony formation, flow cytometry, wound healing, and transwell assays. The effects of GIMAP7 on the extracellular acidification rate (ECAR), oxygen consumption rate (OCR), lactate production, and glucose uptake were evaluated. In addition, the related mRNA and protein expression was detected employing immunohistochemical, western blot, and qRT‐PCR. A xenograft tumor model was established in nude mice to evaluate the effects of GIMAP7 on tumor growth.

**Results:**

GIMAP7 was lowly expressed in LUAD tissues and cells. GIMAP7 inhibited the proliferation, mobility, EMT, glycolysis, but promoted apoptosis in LUAD cells. Moreover, we also confirmed that GIMAP7 suppressed Smo/AMPK signaling in LUAD cells. By adding the Smo agonist SAG and AMPK agonist GSK621, the results of rescue experiments further verified that GIMAP7 played a role in LUAD inhibition through inhibition of the Smo/AMPK signaling pathway. Furthermore, the role of GIMAP7 in inhibiting tumor growth was verified in vivo.

**Conclusions:**

These results demonstrate that GIMAP7 could inhibit cell proliferation, mobility and glycolysis, but accelerate apoptosis via repressing the Smo/AMPK signaling pathway in LUAD.

## Graphical abstract

High expression of GIMAP7 represses the Smo/AMPK signaling pathway in lung adenocarcinoma cells. The inactivated Smo/AMPK signaling pathway further alters gene transcription to inhibit lung adenocarcinoma cell epithelial‐mesenchymal transition and glycolysis.

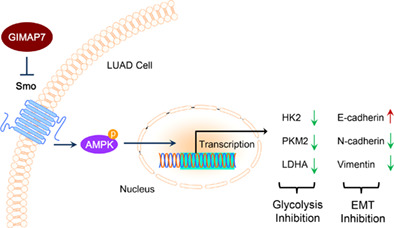

## INTRODUCTION

Lung cancer, with the highest incidence of cancer in both men and women, is the leading cause of cancer related deaths worldwide.^1^ Lung adenocarcinoma (LUAD) is the most common type of lung cancer.^2^ Although there are currently ongoing advances in surgical, medical, and radiation treatments, LUAD remains one of the most rapidly lethal malignancies with an overall survival of less than 5 years.^3^, ^4^ Hence, the development of LUAD treatment needs to be further studied, especially to search for new molecular mechanisms and effective therapeutic targets.

The GTPase immunity‐associated nucleotide‐binding proteins (GIMAPs) family consist of seven functional genes in human, including GIMAP1, GIMAP4, GIMAP5, GIMAP6, GIMAP7, GIMAP8, and GIMAP9.^5^, ^6^, ^7^ GIMAP family members have been considered to be involved in the progression of malignancies by modulation of the immunologic microenvironment.^8^, ^9^, ^10^ In recent years, growing studies have demonstrated that GIMAP7 expression is downregulated in a series of malignancies and is closely associated with the development and progression of many malignancies.^11^, ^12^ Usman et al.^13^ have reported that, in the serum of patients with oral squamous cell carcinoma, GIMAP7 was lowly expressed. The expression level of GIMAP7 was verified to be dramatically low in breast tumor tissues.^11^ In endometrial cancer, a high level of GIMAP7 has been reported to be beneficial in disease‐free and overall survival.^14^ Interestingly, the underexpression of GIMAP7 has been observed in LUAD tissues.^15^ Nevertheless, the functional effects as well as the molecular mechanism of GIMAP7 in LUAD are poorly understood.

Therefore, in the present study, we explored the functions of GIMAP7 in LUAD progression and the potential mechanisms. Our findings revealed that GIMAP7 could inhibit cell proliferation, mobility, and glycolysis, and accelerate apoptosis via repressing Smoothened (Smo) receptor/AMP‐activated protein kinase (AMPK) signaling in LUAD cells. Additionally, we demonstrated that the upregulation of GIMAP7 could prevent LUAD progression in vivo.

## METHODS

### Bioinformatics

Analysis of GIMAP7 expression in LUAD was performed via The Cancer Genome Atlas database (TCGA, https://cancergenome.nih.gov/). Moreover, we utilized TCGA and GEPIA (http://gepia2.cancer-pku.cn) databases for analyzing clinical data associated between GIMAP7 expression and LUAD. Kaplan–Meier plots presents overall survival of LUAD patients. Gene set enrichment analysis (GSEA, http://software.broadinstitute.org/gsea/index.jsp) was used to gain further insight into the signaling pathways related to GIMAP7 expression in LUAD.

### Next‐generation sequencing

Total RNA was collected from A549 cells transfected with GIMAP7 siRNAs or the control siRNA via utilizing TRIzol (Invitrogen). Sequencing libraries preparation and RNA‐sequence experiments were performed by Novogene. After the generation of the libraries, the clustering of the libraries was sequenced by the Illumina NovaSeq 6000. The image data were converted into reads using CASAVA. Clean reads were aligned with the reference genome utilizing Hisat2 (version 2.0.5). After that, FeatureCounts (version 1.5.0‐p3) was used to count the number of reads as well as calculate the fragments per kilobase of transcript per million fragments mapped (FPKM). Differential expression analysis was then conducted utilizing DESeq2 R package (1.20.0).

### Clinical sample collection

Paraffin‐embedded cancer tissue samples and paired adjacent tissues were obtained from 30 patients with LUAD admitted to our hospital from January 2020 to January 2022. None of the patients were subjected to radiotherapy, chemotherapy, or immunotherapy before the tumor removal. The collection of LUAD tissues for research purposes was approved by the relevant human research ethics committees of our hospital. All the tissue samples were collected after obtaining written consent from all patients with LUAD.

### Cell culture and transfection

Human bronchial epithelioid cell line 16HBE and lung cancer cell lines (H1650, A549, PC9 and H1299) were cultured in RPMI 1640 medium (Thermo Fisher) supplemented with 10% fetal bovine serum (FBS: Thermo Fisher) at 37°C. The 16HBE and PC9 cells were purchased from Cobioer Biosciences, while H1650, A549 and H1299 cells were supplied by Procell.

The pcDNA‐GIMAP7 plasmid, a GIMAP7 overexpression plasmid obtained from FulenGen, was transfected into H1650 cells using lipofectamine 3000 (Invitrogen). At the same time, small interfering RNAs against GIMAP7 (si1‐GIMAP7 and si2‐GIMAP7) and si‐NC were obtained from Tsingke and transfected into A549 and H1299 cells using lipofectamine 3000 (Invitrogen). Cancer cells were harvested 48 h after transfection and used for the following functional experiments. The sequence of siRNAs are listed as follows: si1‐GIMAP7: 5'‐GGCATAGGTTTTTGTCGAA‐3'; si2‐GIMAP7: 5'‐GACACCACCTGCAAGGAAA‐3'; si‐NC: 5'‐TTCCTCGAACGTGTCACTG‐3′.

### Cell counting kit‐8 (CCK8) assay

At 48 h after transfection, the gene‐transfected LUAD cells (3 × 10^3^ cells/well) were cultured in a 96‐well plate at 37°C. The optical density value (OD_450_) was measured 2 h after the addition of CCK‐8 reagent (Dojindo) at 0, 24, 48, 72, and 96 h by employing a microplate reader (Thermo Fisher).

### 5‐ethynyl‐20‐deoxyuridine (EdU) incorporation assay

An EdU assay kit (Beyotime) was utilized to assess the proliferation rate of H1650, A549, and H1299 cells as previously described.^16^ The cells were then cultured at 37°C with EdU reagent for 2 h prior to fixation with 4% formaldehyde for 30 min, 0.3% Triton X‐100 permeabilization for 10 min. Afterwards, DAPI was applied to counterstain the cell nucleus for 15 min. Finally, cells were visualized with a fluorescence microscope (Nikon).

### Colony formation assay

After transfection, the cells (H1650, A549 and H1299) were digested and the suspension of H1650, A549 and H1299 cells (1 × 10^3^ cells/well) inoculated in a six‐well plate. After 14 days of culture at 37°C, the cancer cells were dyed utilizing 0.1% crystal violet, washed, imaged, and the colony number was counted.

### Apoptosis assay

Apoptosis assay was carried on a BD FACSCalibur flow cytometer (BD Biosciences) utilizing the Annexin V‐fluorescein isothiocyanate (FITC) apoptosis detection kit with Annexin V‐FITC and propidium iodide based on the manufacturer's instructions (Beyotime).

### Wound healing assays

The transfected H1650, A549, and H1299 cells were implanted into six‐well plates. Once cells formed a tight cell monolayer, the monolayer was scratched utilizing a 200‐μL pipette tip, with at least three lines crossing each well. Subsequently, the cells were cultured in serum‐free DMEM at 37°C for 24 h. A microscope (Nikon) was used for imaging at 0 and 24 h.

### Transwell assay

The cell migration and invasion assays were carried out utilizing the transwell 24‐well Boyden chamber (Corning) with an 8‐μm pore. The transfected H1650, A549, and H1299 cells, suspended in serum‐free RPMI 1640 culture medium, were seeded into the upper chamber coated with Matrigel (for invasion, Corning) or without Matrigel (for migration, Corning), and culture medium containing 10% FBS was added into the lower chamber. Following 24 h of cultivation, H1650, A549 and H1299 cells on the lower membrane surface were fixed using 4% formaldehyde for 30 min and then stained using a 0.1% solution of crystal violet for 20 min. Finally, the number of migrating or invasive cells was counted under a light microscope.

### Mitochondrial stress and glycolysis stress tests

Extracellular acidification rate (ECAR) or oxygen consumption rate (OCR) was analyzed utilizing Seahorse XF glycolysis stress test kit and the Seahorse XF cell Mito stress test kit (Agilent) on the Seahorse XFe24, respectively, as previously described.^15^

### Lactate production and glucose consumption measurement

The concentration of lactate production and glucose consumption in H1650, A549 and H1299 cells was respectively detected by using the lactate assay kit (Abcam, UK) and the glucose kit (Solarbio), based on the manufacturer's instructions.

### Quantitative reverse transcription‐polymerase chain reaction (RT‐PCR)

Total RNAs were collected from LUAD tissues or cells with TRIzol (Beyotime). Afterwards, complementary DNA was synthesized applying a Prime Script RT‐PCR kit (Takara), followed by quantification by the SYBR Super Real PreMix Plus (Takara). GAPDH was used for normalization. The sequences of primers were: GIMAP7, F: 5’‐TCAACTTGTTCAAGAGA AGGTCT‐3' and R: 5'‐ACCAGAACGATCCTCAGGGA‐3′; and GAPDH, F: 5'‐TTAGGAAAGCCTGCCGGTGA‐3' and R: 5'‐GCCCAATAC GACCAAATCA GAGAA T‐3′.

### Western blot analysis

For protein extraction, human LUAD tissues, LUAD cells and mice xenograft tumor tissues were homogenized in ice‐cold radioimmunoprecipitation assay (RIPA) buffer (Beyotime). Protein concentration was quantified utilizing the bicinchoninic acid kit (Beyotime) using bovine serum albumin (BSA) as a standard. The 10% polyacrylamide gel was loaded with 50 μg protein, electrophoresed at 180 V, and electrotransferred onto polyvinylidene fluoride (PVDF) membranes. Afterwards, these membranes were treated in 5% BSA in tris‐buffered saline with tween 20 (TBST) and then incubated with primary antibodies to GIMAP7 (Proteintech), E‐cadherin (Proteintech), N‐cadherin (Proteintech), vimentin (Abcam), hexokinase II (HK2, Abcam), PKM2 (Cell Signaling), lactate dehydrogenase (LDHA: Cell Signaling), Smo (Proteintech), p‐AMPK (Abcam), AMPK (Abcam) and glyceraldehyde 3‐phosphate dehydrogenase (GAPDH: Proteintech) at 4°C overnight. Afterwards, we incubated the membrane in the secondary antibody (Proteintech) for 60 min. At last, blot was developed by ECL (Beyotime).

### Establishment of tumor xenografts in mice

BALB/C nude mice (6–8 weeks) were supplied by GemPharmatech and housed in individually ventilated cages and under specified pathogen‐free conditions. The H1650 cells (1 × 10^7^), which were transfected with a GIMAP7 overexpression lentivirus vector or a control lentivirus vector, were subcutaneously inoculated directly into the right dorsal flank per mouse. Tumor growth was measured from day 12 to day 30 using calipers every 3 days. Mice were sacrificed 30 days after injection, and then xenografted tumor tissues were collected and weighed.

### Histology and immunohistochemistry

Human LUAD tissues and mouse xenografts tumor tissues were fixed in 4% paraformaldehyde for 24 h and then sectioned into 5‐μm thickness. Afterwards, the slide was dewaxed, rehydrated, and incubated in 3% H_2_O_2_ for 10 min at 37°C. After antigen retrieval, the slides were blocked using 3% BSA, and were then incubated overnight at 4°C in primary antibodies: anti‐GIMAP7 (Proteintech) and anti‐Ki67 (Abcam). Following washing, the slides were incubated with a secondary antibody for 30 min, colored by diaminobenzidine solution (Zhong Shan Jin Qiao), and then restained with hematoxylin (Sigma). Representative images were taken utilizing a light microscope. For TUNEL staining, a TUNEL staining kit (Beyotime) was applied to determine the number of apoptotic cells within each section based on the manufacturer's protocol. Finally, the Ki67‐ and TUNEL‐positively stained cells were counted under a fluorescence microscope from five randomly selected fields.

### Statistical analysis

Statistical analyses were carried out using SPSS 22.0 software. Data are expressed as mean ± SD. Student's *t*‐test and one‐way ANOVA were adopted for statistical analyses. Statistical significance was defined as *p* < 0.05.

## RESULTS

### GIMAP7 is lowly expressed in LUAD

**FIGURE 1:**
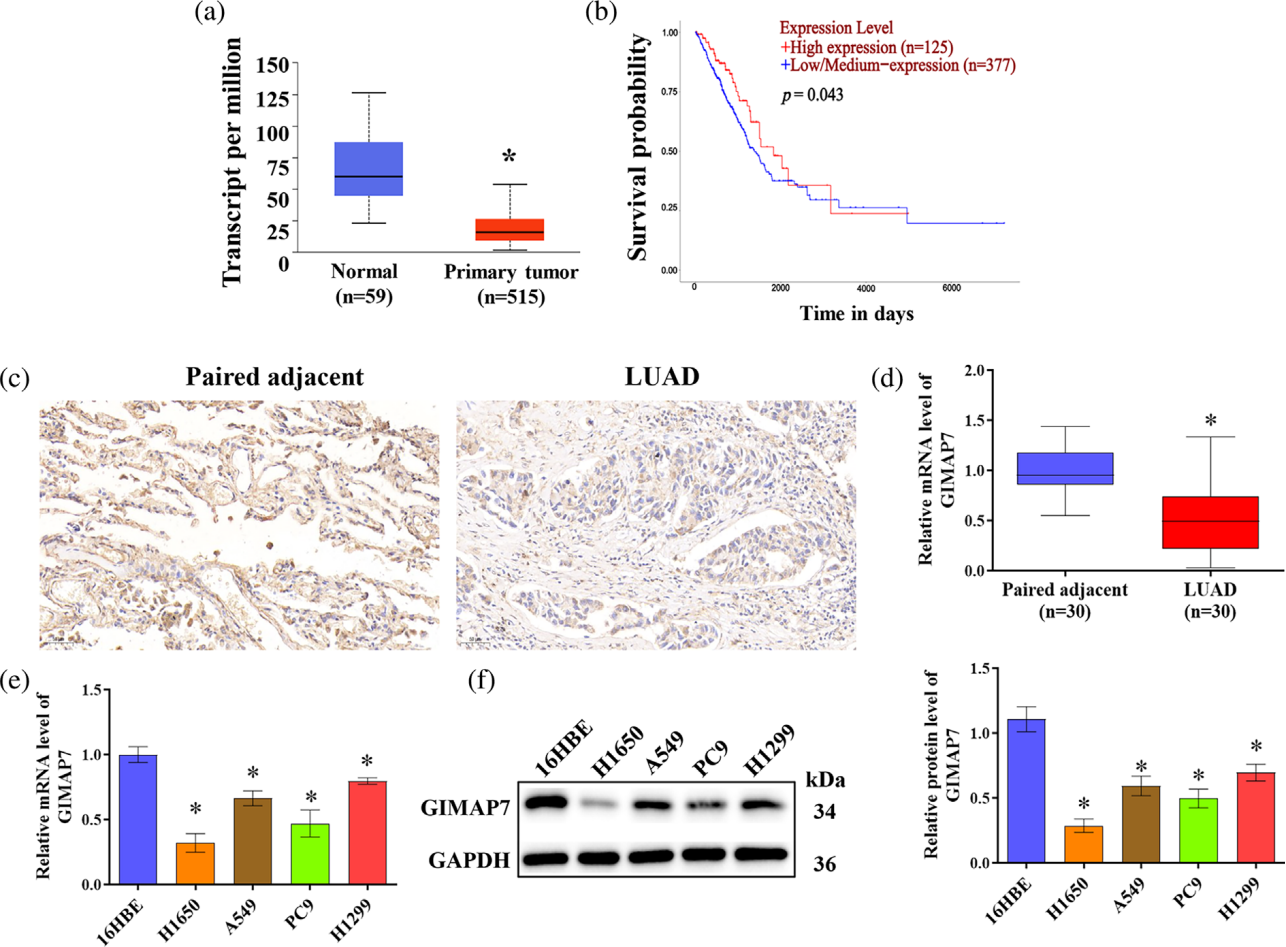
GIMAP7 was lowly expressed in lung adenocarcinoma (LUAD). (a) GIMAP7 level in normal (*n* = 59) and LUAD (*n* = 515) samples in the TCGA dataset. (b) Kaplan–Meier survival plots of overall survival based on GEPIA was analyzed using long‐rank test. (c) Representative images of GIMAP7 immunohistochemistry staining. (d) GIMAP7 expression in 30 pairs of LUAD and adjacent tissues was tested utilizing quantitative reverse transcription‐polymerase chain reaction (qRT‐PCR). (e and f) GIMAP7 expression in 16HBE and four LUAD cell lines (H1650, A549, PC9, and H1299) was evaluated using qRT‐PCR and western blot. Data in (a, d) was analyzed using the unpaired *t*‐test. Data in (e, f) was analyzed using the one‐way analysis of variance (ANOVA). *p < 0.05.

The data from the TCGA database manifested that GIMAP7 was lowly expressed in LUAD tissues as compared to the normal controls (Figure 1a). In addition, low GIMAP7 level was associated with a poor clinical outcome in patients with LUAD (Figure 1b). Further in‐depth analysis of the 30 paired LUAD samples using immunohistochemistry and qRT‐PCR demonstrated that the expression of GIMAP7 was low in LUAD tissues (Figure 1c,d). Additionally, GIMAP7 was also lowly expressed in LUAD cell lines (H1650, A549, PC9, and H1299) when compared to normal bronchial epithelioid cells (16HBE) (Figure 1e,f).

### GIMAP7 inhibits proliferation, mobility, but promoted apoptosis in LUAD cells

**FIGURE 2:**
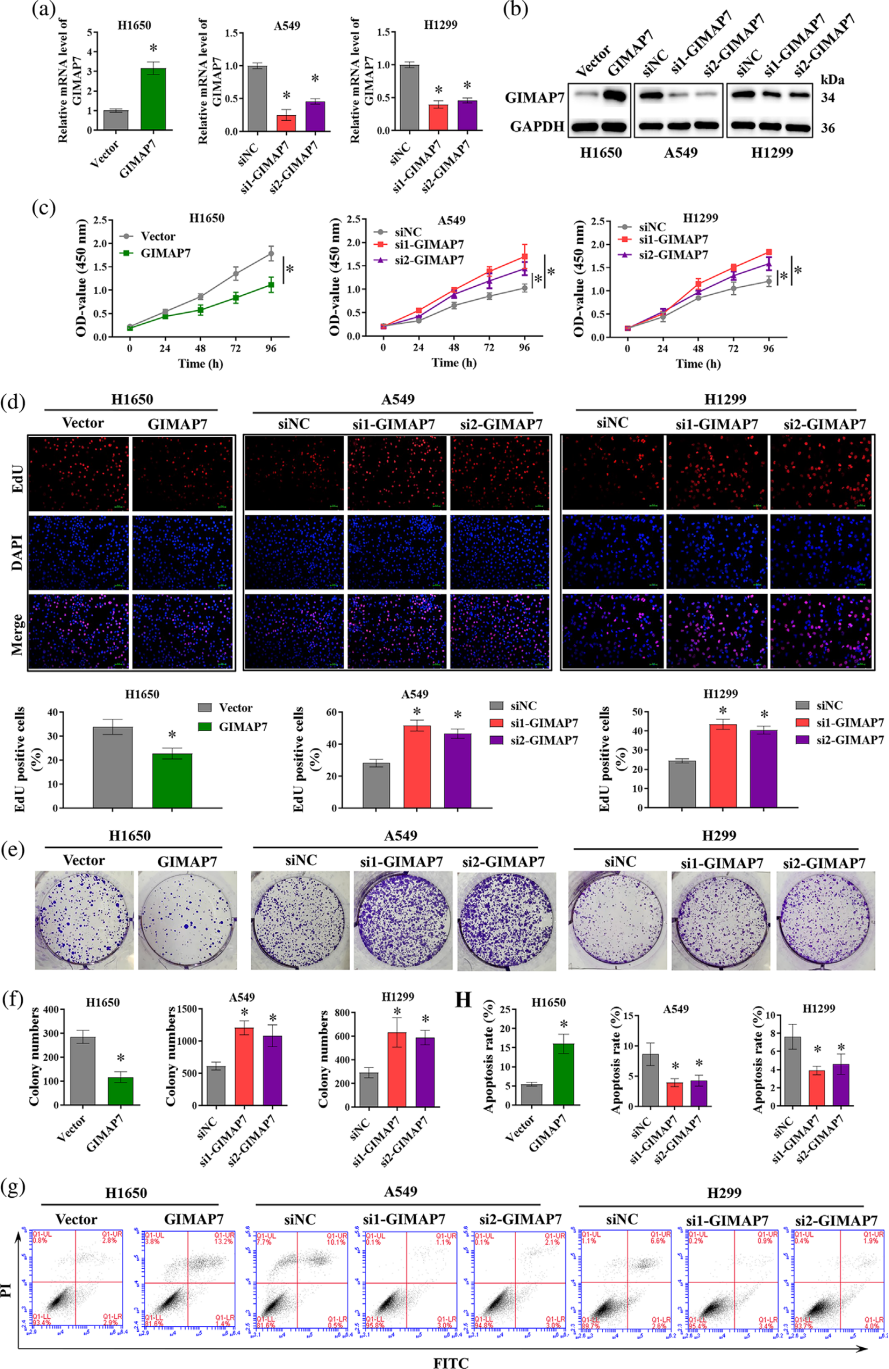
GIMAP7 inhibited the proliferation but promoted apoptosis in lung adenocarcinoma (LUAD) cells. (a) Quantitative reverse transcription‐polymerase chain reaction (qRT‐PCR) and (b) western blot analysis of GIMAP7 expression in H1650 cells transfected with pcDNA‐GIMAP7 overexpression plasmids, or the A549 and H1299 cells transfected with GIMAP7 specific siRNAs. (c) Cell counting kit‐8 (CCK‐8), (d) 5‐ethynyl‐20‐deoxyuridine (EdU), and (e, f) colony formation assays were applied to test the proliferation of LUAD cell lines. (g, h) Flow cytometry was used to test LUAD cell apoptosis. Data in (a, d, e, f, g, h) was analyzed using the unpaired *t*‐test or one‐way analysis of variance (ANOVA). Data in (c) was analyzed using two‐way ANOVA. *p < 0.05.

**FIGURE 3:**
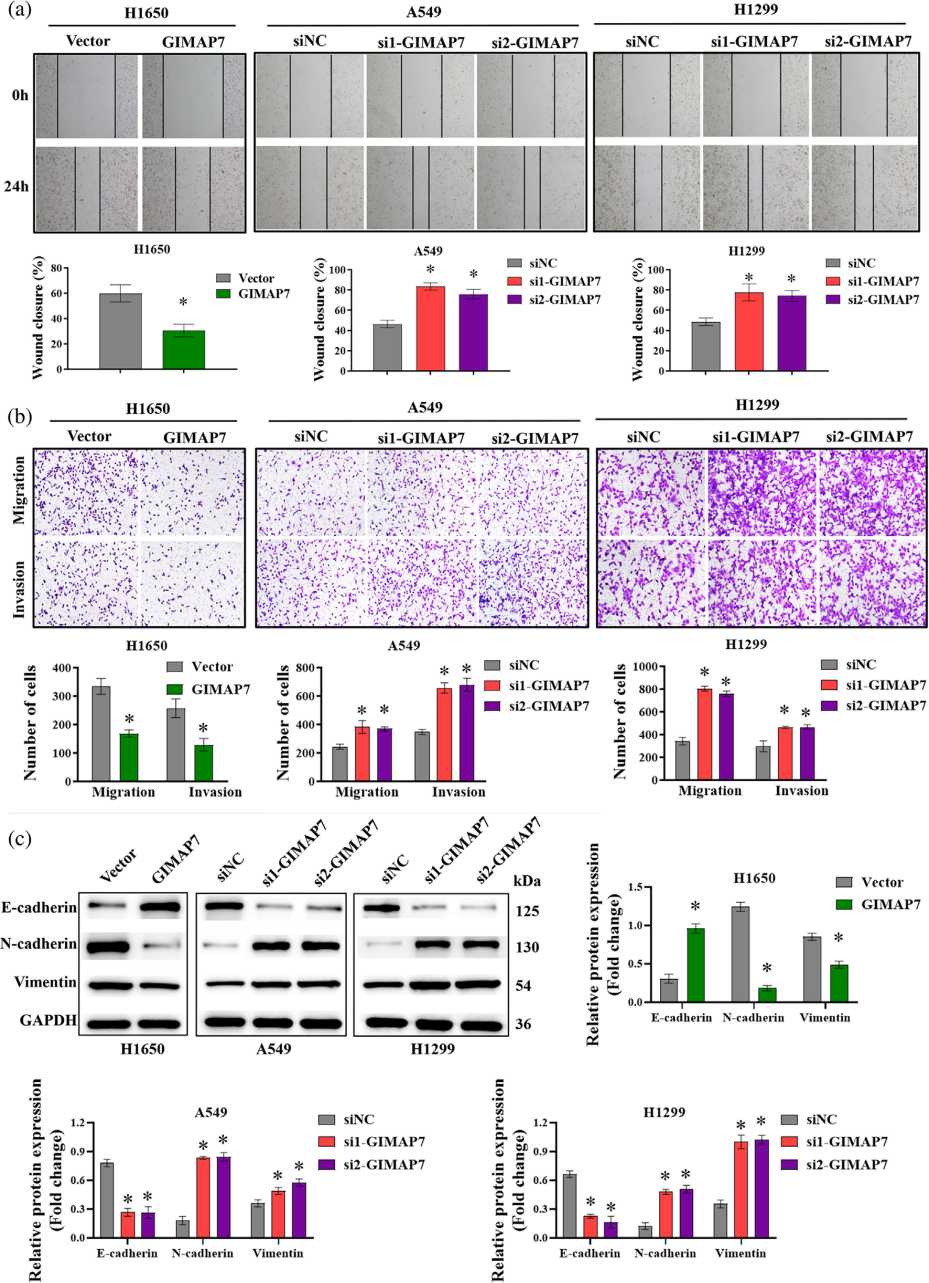
GIMAP7 inhibited cell migration and invasion, as well as epithelial‐mesenchymal transition (EMT) in lung adenocarcinoma (LUAD) cells. Migration and invasion abilities of H1650, A549 and H1299 cells with GIMAP7 overexpression or GIMAP7 silencing were measured employing (a) wound healing and (b) transwell assays. (c) The expression of epithelial marker E‐cadherin, mesenchymal markers, vimentin and N‐cadherin, were analyzed utilizing western blot. Data was analyzed using the unpaired *t*‐test or one‐way analysis of variance (ANOVA). *p < 0.05.

To assess the biological effects of GIMAP7 on LUAD, we overexpressed GIMAP7 in H1650 cells and silenced GIMAP7 in A549 and H1299 cells. Then, the transfection efficiency was determined by qRT‐PCR (Figure 2a) and western blot analysis (Figure 2b). After confirming the transfection efficiency, we determined the effects of GIMAP7 on LUAD cell proliferation using CCK‐8, colony formation, and EdU assays. All results showed that the upregulation of GIMAP7 reduced cell proliferation of LUAD cells, while the downregulation of GIMAP7 elevated cell proliferation (Figure 2c–f). At the same time, Figure 2g,h data demonstrated that apoptosis was enhanced by GIMAP7 upregulation but was repressed by lowering GIMAP7 expression in LUAD cells. Cell migration and invasion were investigated via wound healing and transwell assays. The upregulation of GIMAP7 in H1650 cells effectively decreased the migrating and invasive capacities, while the downregulation of GIMAP7 in A549 and H1299 cells caused a significant increase of the migrating and invasive capacities (Figure 3a,b). The epithelial‐mesenchymal transition (EMT) can enable stationary epithelial cell to acquire the capacity to migrate and invade as a single cell.^16^ As seen in Figure 3c, the upregulation of GIMAP7 caused a decrease of N‐cadherin and vimentin protein levels, but an increase of E‐cadherin in LUAD cells. On the contrary, the downregulation of GIMAP7 led to a significant increase of N‐cadherin and vimentin levels, but a decrease of E‐cadherin level in LUAD cells (Figure 3c). These data suggest that GIMAP7 might repress the proliferation, mobility, but accelerate apoptosis in LUAD cells.

### GIMAP7 inhibits glycolysis in LUAD cells

**FIGURE 4:**
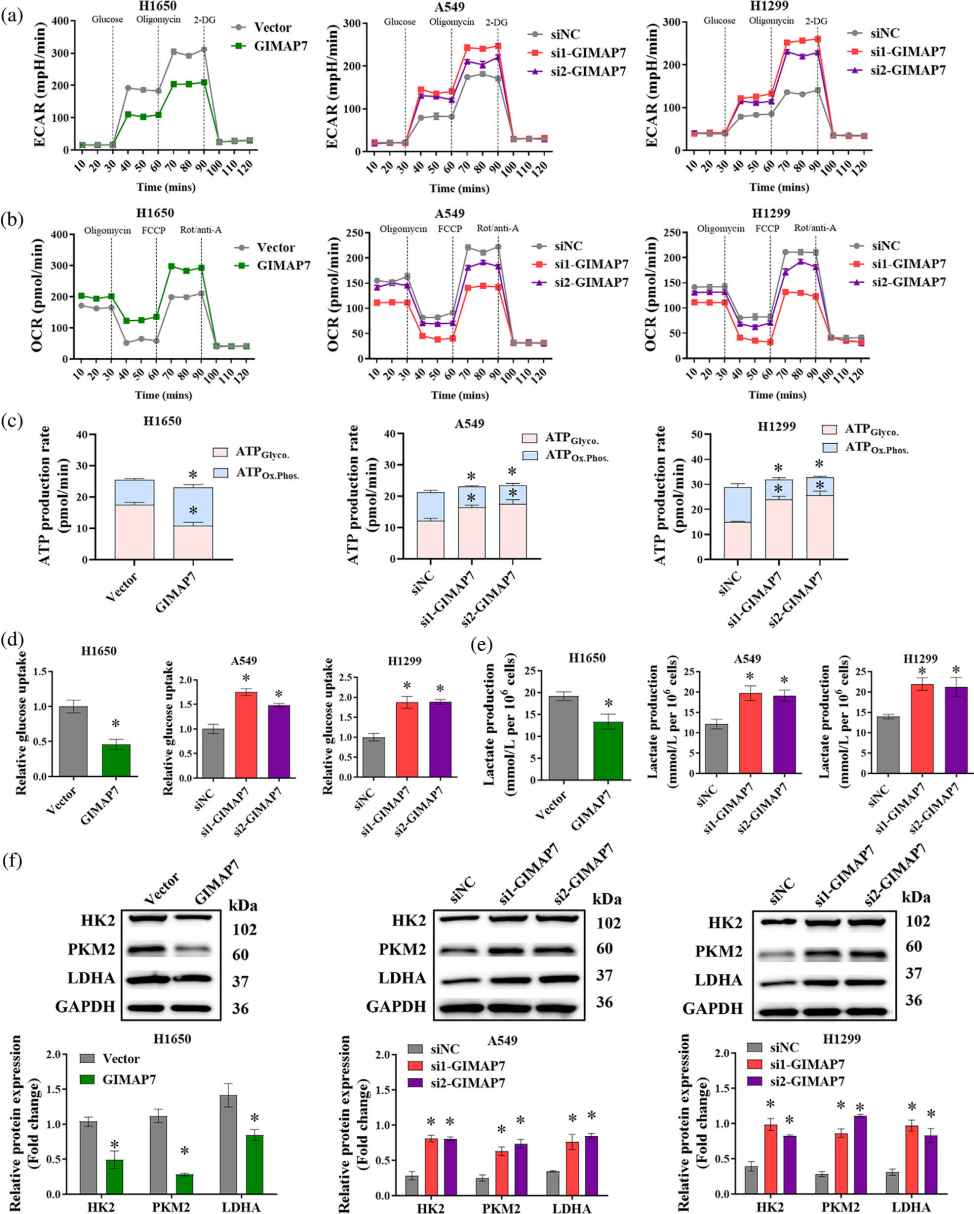
GIMAP7 inhibited the glycolysis of lung adenocarcinoma (LUAD) cells. (a and b) The effects of GIMAP7 on the extracellular acidification rate (ECAR) and oxygen consumption rate (OCR) of LUAD cells. (c) Adenosine triphosphate (ATP) production rate from oxidative phosphorylation (ATP_Ox.Phos_., blue) and glycolysis (ATP_Glyco._, pink) in LUAD cells. (d and e) The effects of GIMAP7 on the glucose uptake and lactate production of LUAD cells. (f) The expression of glycolysis markers HK2, PKM2, and LDHA were analyzed utilizing western blot. Data was analyzed using the unpaired *t*‐test or one‐way analysis of variance (ANOVA). *p < 0.05.

As shown in Figure 4a,b, in H1650 cells with GIMAP7 overexpression, ECAR was lower and OCR was higher, which suggested a slower glycolytic rate. At the same time, in A549 and H1299 cells with lowered GIMAP7 expression, ECAR was higher and OCR was lower, which suggested a higher glycolytic rate (Figure 4a,b). In addition, the adenosine triphosphate (ATP) production rate from oxidative phosphorylation (ATP_Ox.Phos._) was higher and the ATP production rate from glycolysis (ATP_Glyco._) was lower in H1650 transfected with GIMAP7 overexpression plasmid than those transfected with empty vector. In A549 and H1299 cells, transfection with GIMAP7 specific siRNAs led to a lower ATP_Ox.Phos._ and a higher ATP_Glyco._ than cells transfected with siNC (Figure 4c). The glucose uptake and lactate production were decreased by the upregulation of GIMAP7 but increased by the downregulation of GIMAP7 in LUAD cells (Figure 4d,e). Then, we investigated glycolysis markers in H1650 cells with GIMAP7 overexpression, as well as in A549 and H1299 cells with lowering GIMAP7 expression. The results manifested that the expression of HK2, PKM2, and LDHA was repressed by the upregulation of GIMAP7, but enhanced by the downregulation of GIMAP7 (Figure 4f).

### GIMAP7 suppresses the Smo/AMPK signaling in LUAD cells

**FIGURE 5:**
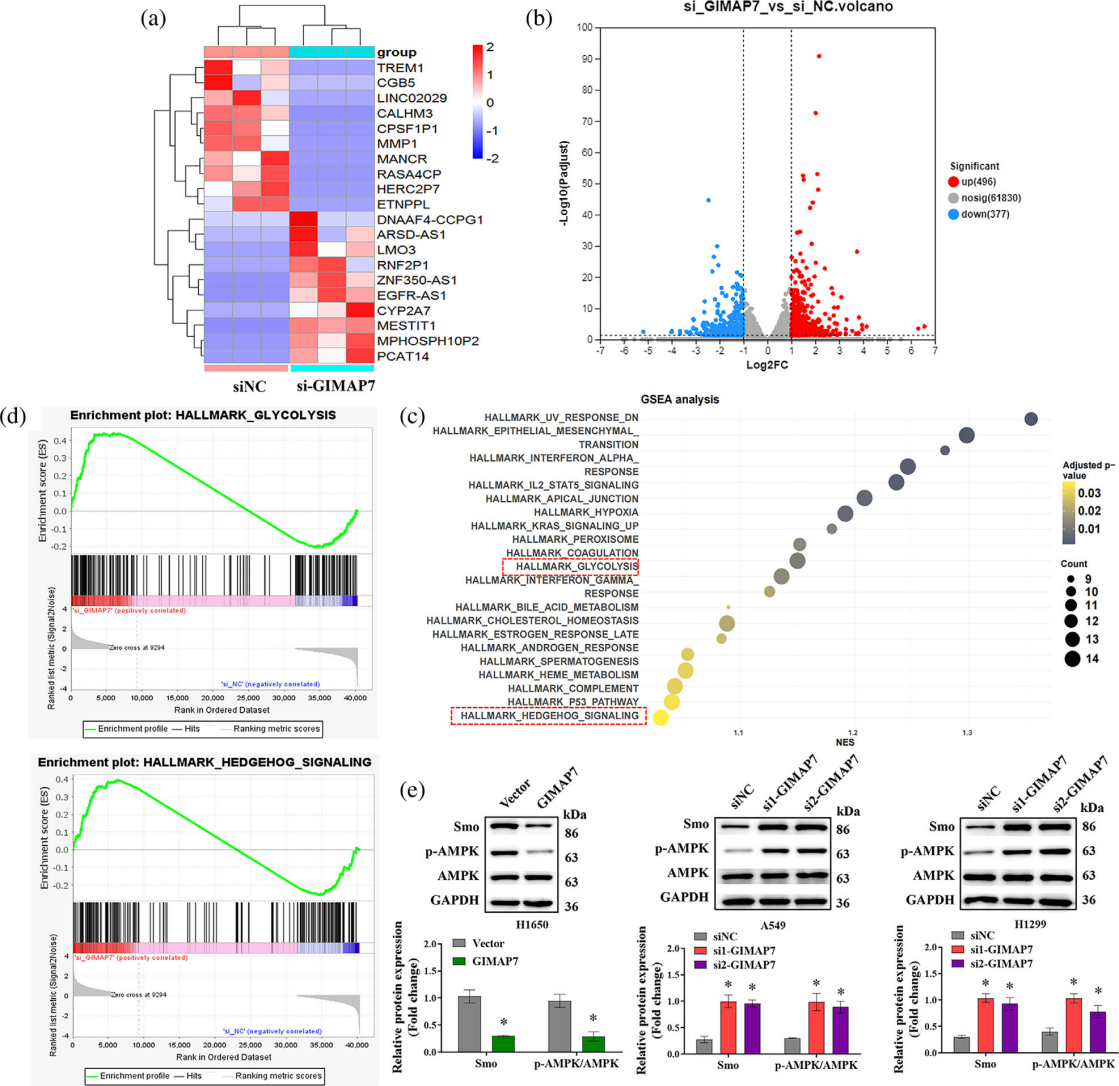
GIMAP7 suppressed the Smo/AMPK signaling pathway in lung adenocarcinoma (LUAD) cells. (a) The differential expression of genes between si‐GIMAP7 and si‐NC cell lines was analyzed using next‐generation sequencing and shown in a heat map. (b) Volcano plots of differentially expressed genes (red points represent the upregulated genes and those in blue represent the downregulated genes). (c and d) Gene set enrichment analysis was used to analyze the GIMAP7‐related signaling pathways in LUAD. (e) Expression of Smo, p‐AMPK, and AMPK in LUAD cells were analyzed utilizing western blot. Data was analyzed using the unpaired *t*‐test or one‐way analysis of variance (ANOVA). *p < 0.05.

To further identify the function of GIMAP7 in modulating LUAD, next‐generation sequencing was performed, and the results illustrated that GIMAP7 silencing caused the dysregulation of 873 genes (496 upregulated and 377 downregulated) (Figure 5a,b). Subsequently, GSEA analysis demonstrated that GIMAP7 was negatively correlated with hedgehog signaling and glycolysis (Figure 5c,d). By using western blot analysis, we further verified that Smo and p‐AMPK levels in LUAD cells was notably decreased by GIMAP7 overexpression, but dramatically elevated by the downregulation of GIMAP7 (Figure 5e). These data reveal that GIMAP7 might suppress the glycolysis and Smo/AMPK signaling pathway in LUAD cells.

### GIMAP7 inhibits cell proliferation, mobility, and glycolysis via repressing Smo/AMPK signaling in LUAD cells

**FIGURE 6:**
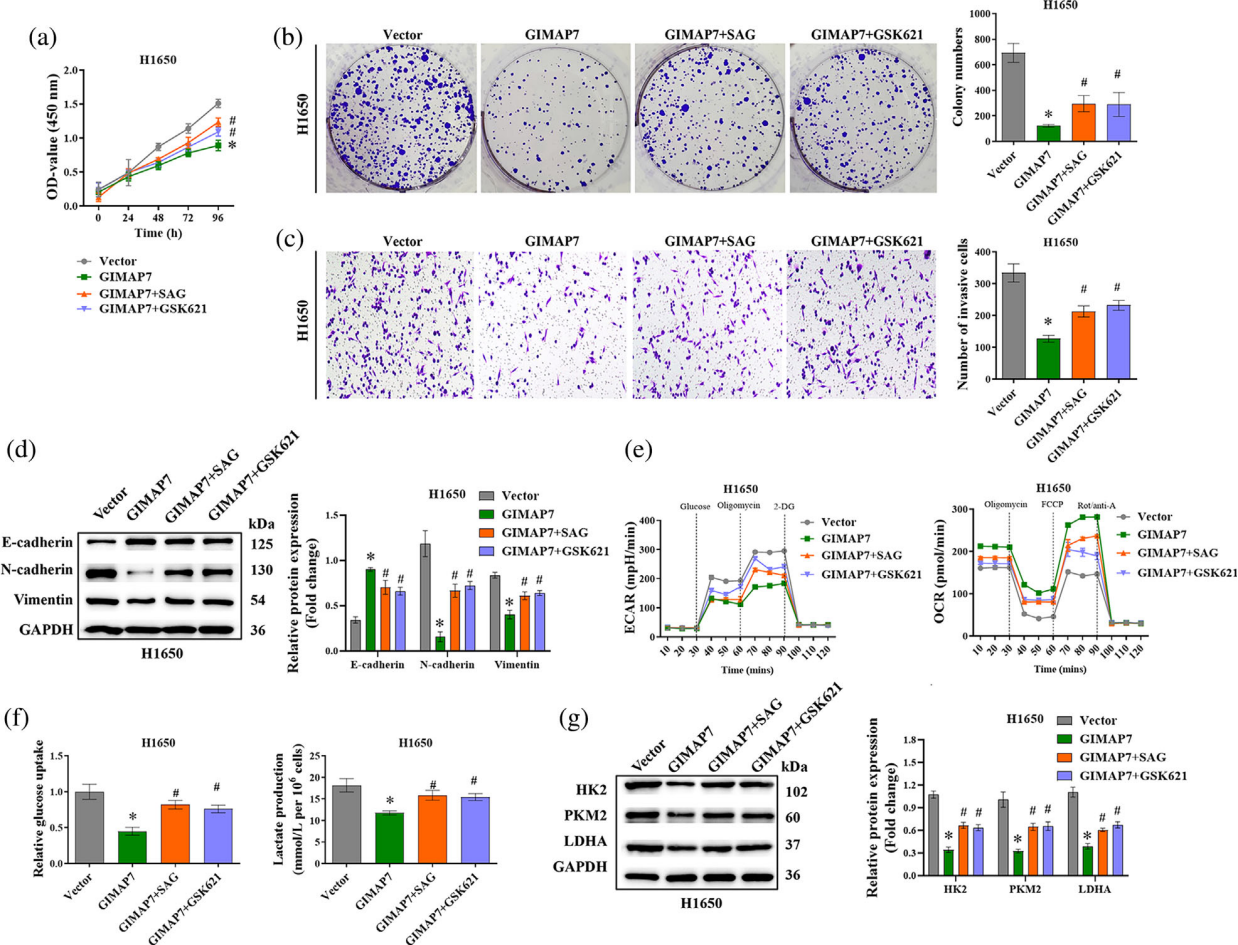
GIMAP7 inhibited cell proliferation, mobility, and glycolysis via repressing the Smo/AMPK signaling pathway in lung adenocarcinoma (LUAD) cells. After transfection with pcDNA‐GIMAP7 overexpression plasmids and the treatment of SAG or GSK621, cell proliferation was tested using (a) cell counting‐kit 8 (CCK‐8) and (b) colony formation assays. (c) The invasive ability was tested utilizing transwell assay. (d) Epithelial‐mesenchymal transition (EMT)‐related protein levels were determined employing western blot. (e) ECAR and OCR in H1650 cells were analyzed employing Seahorse flux analyzer. (f) Glucose uptake and lactate production in H1650 cells were measured using commercial kits. (g) The expression of HK2, PKM2 and LDHA were analyzed utilizing western blot. Data in (a) was analyzed using the two‐way analysis of variance (ANOVA). Data in (b, c, d, f, g) was analyzed using the one‐way ANOVA. *p < 0.05.

To verify whether GIMAP7 inhibits LUAD cell proliferation, mobility, and glycolysis via repressing the Smo/AMPK signaling pathway in H1650 cells, Smo agonist SAG and AMPK agonist GSK621 were added to the culture medium. As seen in Figure 6a–c, the repressive effects of GIMAP7 overexpression on the proliferation and invasion of LUAD cells were significantly reversed by adding SAG or GSK621. Moreover, western blot analysis results confirmed that the higher expression of E‐cadherin and the lower expression of N‐cadherin and vimentin, which were induced by GIMAP7 overexpression, were dramatically abolished by the addition of SAG or GSK621 (Figure 6d). Additionally, the low‐level of ECAR and the high‐level of OCR induced by GIMAP7 overexpression in H1650 cells were reversed by SAG or GSK621 (Figure 6e). At the same time, the decreased glucose uptake and lactate production caused by the high expression of GIMAP7 were significantly reversed by SAG or GSK621 (Figure 6f). In addition, the lower expression of HK2, PKM2 and LDHA induced by GIMAP7 overexpression were significantly abolished by SAG or GSK621 (Figure 6g). These findings suggest that GIMAP7 might suppress the proliferation, mobility, and glycolysis of LUAD cells via repressing the Smo/AMPK signaling pathway.

### GIMAP7 inhibits tumor growth by suppressing the Smo/AMPK signaling pathway in xenograft mouse model

**FIGURE 7:**
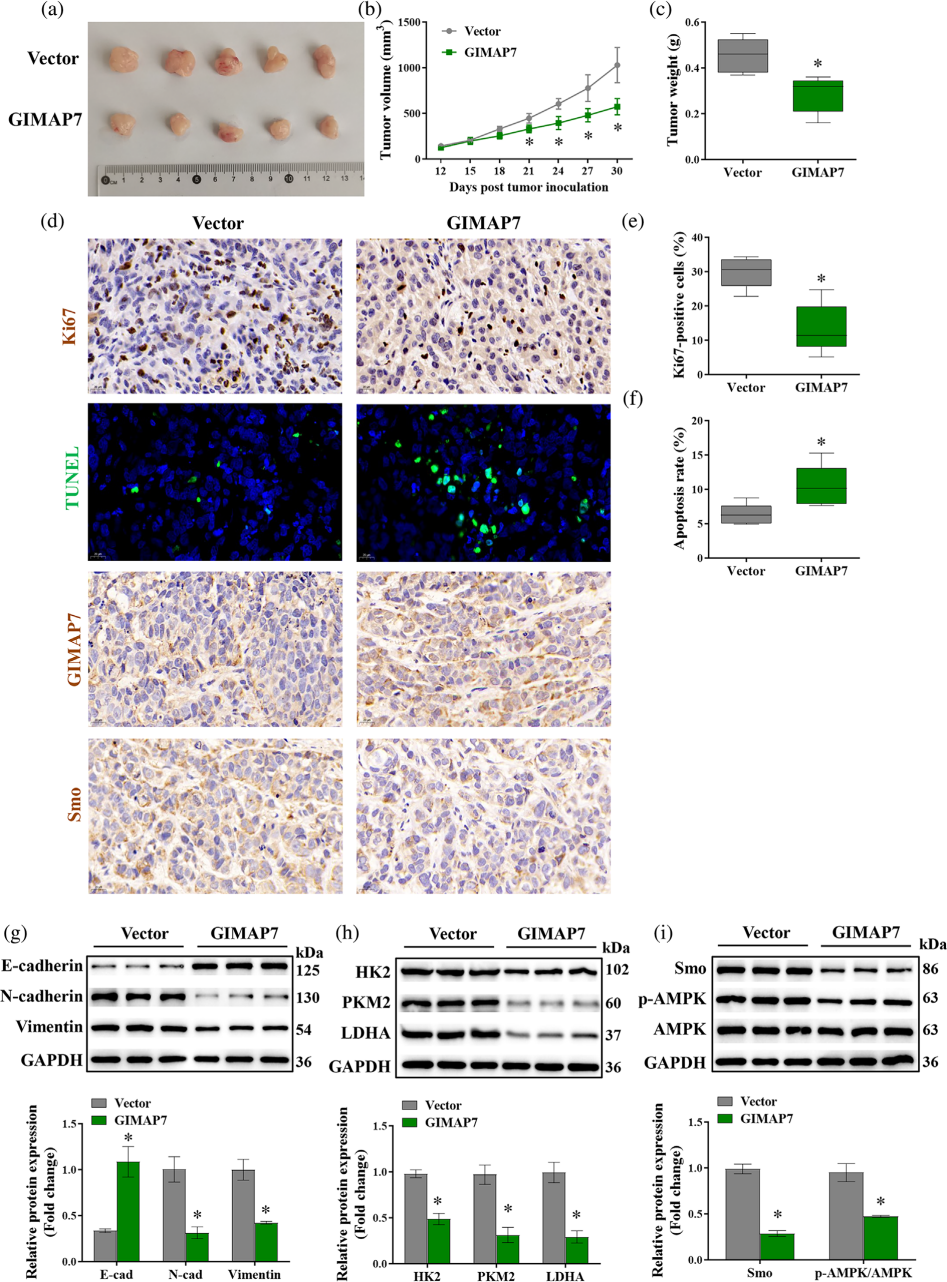
GIMAP7 inhibited tumor growth by suppressing the Smo/AMPK signaling pathway in a xenograft mouse model. (a) Images of xenografts from mice injected with H1650 cells which were pretransfected with GIMAP7 overexpression lentivirus particles or the empty vector. (b) Tumor volume and (c) weight. (d–f) Immunohistochemistry staining of Ki67, GIMAP7, Smo and TUNEL staining in the xenograft tumor. (g–i) The expression of epithelial‐mesenchymal transition (EMT)‐, glycolysis‐, and Smo/AMPK signaling‐related proteins in the xenograft tumor tissues were detected using western blotting analysis. Data in (b) was analyzed using the two‐way analysis of variance (ANOVA). Data in others was analyzed using the unpaired *t*‐test. *p < 0.05.

Next, a xenograft mouse model was established for confirming the results obtained from in vitro experiments. GIMAP7 overexpression notably reduced the tumor weight and volume (Figure 7a–c). Additionally, the data of immunohistochemistry and TUNEL staining showed that the upregulation of GIMAP7 dramatically reduced Ki67‐ and Smo‐positive cells and notably increased apoptosis (Figure 7d–f). Then, we used western blot analysis to investigate the expression of EMT‐, glycolysis‐ and Smo/AMPK signaling‐related proteins in xenograft tumor tissues. The results showed that the upregulation of GIMAP7 significantly reduced the expression of N‐cadherin, vimentin, HK2, PKM2, LDHA, Smo, and p‐AMPK, but elevated E‐cadherin expression (Figure 7g–i).

## DISCUSSION

The five‐year overall survival rate of patients with LUAD is only 17% because of its high rate of metastasis.^17^, ^18^ Hence, a better understanding of the molecular mechanism of LUAD is needed to propose new therapeutic targets. Our data indicated that GIMAP7 might suppress cell proliferation, mobility, EMT, and glycolysis, but facilitate apoptosis of LUAD cells, and the functional impacts of GIMAP7 on LUAD cells possibly via repressing the Smo/AMPK signaling pathway.

GIMAP7 is considered to participate in the progression of many types of tumor, and its expression is downregulated in most malignancies.^10^, ^11^ By analyzing the TCGA database, Qin et al.,^10^ demonstrated that GIMAP7 was lowly expressed in most tumor tissues, such as bladder, urothelial, breast invasive, and lung squamous cell carcinomas. Another bioinformatic analysis on GIMAP family members indicated that GIMAP7 together with GIMAP1, GIMAP5, GIMAP6, and GIMAP8, are lowly expressed in breast cancer tissues.^11^ In addition, GIMAP7 has been recognized as a putative biomarker in oral squamous cell carcinoma as its expression has been found to be significantly low in the serum of patients with oral squamous cell carcinoma compared with healthy controls.^19^ In the present study, we demonstrated the downregulation of GIMAP7 expression in both LUAD tissues and cell lines and confirmed the association between GIMAP7 low‐expression and poor overall survival. Our data further confirmed the potential of GIMAP7 for use as a cancer prognostic marker in the clinical setting.

Cancer is driven by genetic and epigenetic alterations characterized by excessive proliferation and escape mechanisms, including apoptosis disorders, proliferation, migration, invasion, and angiogenesis.^20^, ^21^ In the present study, the specific functions of GIMAP7 on malignant behavior of LUAD cells were studied. We demonstrated that GIMAP7 could suppress the proliferation, migration, and invasion, but accelerate the apoptosis of LUAD cells. In addition, glycolysis is a specific characteristic of energy metabolism in tumor cells.^22^, ^23^ Glycolysis in tumor cells provides adenosine triphosphate (ATP) in an oxygen‐independent manner which ensures the unlimited growth and proliferation of tumor cells even under diminished nutrient supply.^24^ Increasing evidence has now revealed that altered energy metabolism is an important hallmark of cancers.^25^, ^26^ Glucose metabolism is distinguished by the elevated glucose uptake and aerobic glycolysis in tumor cells.^22^ Moreover, aerobic glycolysis has been reported to provide key intermediate metabolites for tumor growth and to exert an important function on reprogramming the metastatic tumor microenvironment.^15^, ^27^ In this study, the results from next‐generation sequencing and bioinformatic analyses predicted the regulation of GIMAP7 on LUAD cell glycolysis. We confirmed this prediction in vitro as evidenced by the significantly reduced glucose uptake, lactate production, and the downregulation of glycolysis markers, including HK2, PKM2, and LDHA when GIMAP7 was overexpressed.

The hedgehog signaling pathway was first identified in the common fruit fly. Hedgehog signaling is a highly conserved evolutionary pathway of signal transmission from the cell membrane to the nucleus.^28^ The hedgehog pathway is mostly inactive or poorly active in the adult organism. Interestingly, in a series of cancers, including prostate, breast, colon, lung, and hematological malignancies, the aberrant activation of the hedgehog pathway can be observed.^29^, ^30^ Canonical activation of the hedgehog pathway occurs via the binding of hedgehog ligands to PTCH1, which further represses Smo.^31^ Smo can further promote the activation of downstream components of hedgehog signaling.^32^ More and more studies have confirmed that the hedgehog pathway is activated in LUAD.^33^, ^34^, ^35^, ^36^ GSEA analysis demonstrated that GIMAP7 was negatively correlated with hedgehog signaling. Furthermore, Teperino et al.^37^ reported that AMPK is a key downstream pathway for hedgehog signaling which plays a role in glucose metabolism. AMPK is considered to be a cellular energy sensor in a wide variety of tissues.^38^, ^39^ In addition, activated AMPK can enhance glucose and fatty acid uptake.^40^ The AMPK pathway has also been demonstrated to be the major regulator of glycolysis andto be crucial for the metabolic reprogramming in cancer cells.^41^, ^42^ In our study, using western blot analysis, we verified that Smo and p‐AMPK levels in LUAD cells were decreased by GIMAP7 overexpression but elevated by GIMAP7 low expression, suggesting that GIMAP7 suppressed the Smo/AMPK signaling in LUAD. Subsequently, rescue experiments were performed to further verify that GIMAP7 played roles in LUAD through Smo/AMPK signaling by using Smo agonist SAG and AMPK agonist GSK621. In addition, we also confirmed that GIMAP7 could inhibit xenograft tumor growth by suppressing Smo/AMPK signaling in vivo.

In conclusion, our findings demonstrated that GIMAP7 was lowly expressed in LUAD tissues and cell lines. Restoration of GIMAP7 expression in LUAD cells might inhibit the proliferation, mobility, and glycolysis, but accelerate apoptosis via repressing the Smo/AMPK signaling pathway. Therefore, our findings may have uncovered a novel effect of GIMAP7 on LUAD progression and indicated that GIMAP7 may be a new target for the treatment of LUAD.

## AUTHOR CONTRIBUTIONS

Liyuan Cui designed the study and wrote the manuscript; Liyuan Cui, Yumei Shen, Shanzhou Duan and Qifeng Ding performed the research; Yifei Wang, Wentao Yang and Yongbing Chen analyzed data. All authors have read and approved the manuscript.

## FUNDING INFORMATION

National Natural Science Foundation of China (82172076); Jiangsu Key Research and Development Plan (Social Development) Project (BE2020653); Key Scientific Program of Jiangsu Provincial Health Commission (ZD2021033); Gusu Health Leading Talent Program of Suzhou (GSWS2021020); Discipline construction project of the Second Affiliated Hospital of Soochow University (XKTJ‐XK202004); Scientific Program of Suzhou Municipal Health and Health Committee (LCZX202004).

## CONFLICT OF INTEREST STATEMENT

The authors declare that they have no conflicts of interest.

## ETHICS APPROVAL AND CONSENT TO PARTICIPATE

The protocol of this research was approved by the Ethics Committee of the Second Affiliated Hospital of Soochow University. All patients signed their written informed consent. The experimental protocol of the study was performed in accordance with the Guide for the Care and Use of Laboratory Animals. The protocol of this research has been approved by the Ethics Committee of the Second Affiliated Hospital of Soochow University.

## Data Availability

The datasets used and analyzed during the current study are available from the corresponding author on reasonable request.
